# Hill-placement of manure and fertilizer for improving maize nutrient- and water-use efficiencies in the northern Benin

**DOI:** 10.1016/j.heliyon.2023.e17823

**Published:** 2023-07-08

**Authors:** Mouiz W.I.A. Yessoufou, Pierre G. Tovihoudji, Sissou Zakari, André Adjogboto, A. Jonas Djenontin, P.B. Irénikatché Akponikpè

**Affiliations:** Laboratory of Hydraulics and Environmental Modeling (HydroModE Lab), Faculty of Agronomy (UP/FA), University of Parakou, 03 PO Box 351, Parakou, Benin

**Keywords:** Farmyard manure, Internal utilization efficiency, Nutrient management, Recovery efficiency, Sub humid zone

## Abstract

Optimizing the use of organic and mineral fertilizer in rain-fed maize production is crucial for sustainable food production in sub-Saharan Africa. This study investigates the effect of hill-placement of two nutrient sources (farmyard manure and synthetic fertilizer) on nutrient- and water-use efficiencies of maize crops i.e. recovery efficiency (NUEre), internal utilization efficiency (NUEie) and water use efficiency (WUE). A four-year trial was conducted in the tropical sub-humid zone of the northern Benin with a factorial combination of farmyard manure at three levels (0, 3 and 6 t ha^−1^, hereafter NM, 3M and 6M, respectively) and three levels of fertilizer [0% (NF), 50% (50F) and 100% (100F) of the recommended rate (76 kg N + 13.1 kg P + 24.9 kg K ha^−1^) by the national center for agricultural research. The NUEre decreased with increasing rate of manure and/or fertilizer, but the decreasing rate was lower under combined manure and fertilizer application. However, the NUEie increased with the increasing manure and fertilizer amounts. The WUE was significantly higher in 3M and 6M treatments than in NM treatment, and higher in 50F and in 100F than in NF treatments. The combination of 3000 kg ha^−1^ farmyard manure with half recommended fertilizer rate (100 kg ha^−1^) could be suggested as an optimal nutrient management practice for maize production in the Northern Benin. Future studies should target the other agro-ecological zones in Benin, and also consider other widely cultivated crops in the study area for reducing yield gaps and promote food security.

## Highlights

• Maize nutrient use efficiency is optimal under combination of manure and fertilizer.
• Maize water use efficiency increases with rising manure and/or fertilizer rates.
• Manure and fertilizer had correlation with maize nutrient uptake, growth and yields.
• Recommended rate of manure and fertilizer to maize could be reduced by 50%.

## 1 Introduction

Nutrient use efficiency (NUE) and water use efficiency (WUE) have helped to evaluate agricultural productivity in the actual context of soil degradation [1,2] and limited water availability [3,4]. On one hand, NUE is the ability of plants to extract nutrients from soil to produce biomass or grain [5]. Plants require adequate amount of nutrient for their growth, especially nitrogen, phosphorous and potassium. Hence, the improvement of NUE is therefore crucial for sustainable crop intensification. On the other hand, WUE refers to the effectiveness of water utilization by crop [6], and it is expressed as the ratio of biomass or yield by unit water use [7,8]. The ability of plants to tolerate limited water availability is often defined by higher WUE, therefore, improving crops’ WUE is one of the best strategies for attaining food security [9]. This is even more true in Sub Saharan Africa (SSA) due to the influence of climate issues, which can cause prolonged dry spells during growing seasons [10,11].

Several strategies have been investigated for improving nutrient- and water-use efficiencies for better crop productivity; such as application of organic amendments [[12], [13], [14]], fertilizer microdosing [[15], [16], [17]], integrated crop-livestock systems [[18], [19], [20]], conservation agriculture [[21], [22], [23]], and integrated soil fertility management (ISFM) [[24], [25], [26]]. Indeed, research have been implemented in SSA on ISFM that uses the combined application of organic and inorganic amendments for crop intensification [[27], [28], [29]]. For instance, Mutegi et al. [30] stated that the adoption of ISFM in SSA can boost soil health, crop yield and WUE in cereal and legumes farming because of the ability of ISFM in improving soil fertility, soil organic matter and soil water retention. Salama et al. [31] also reported that combining mineral nitrogen and farmyard manure (FYM) results in the higher maize NUE compared to mineral nitrogen alone. However, most studies in SSA highlighted that crop productivity is mainly limited by nutrient management practices rather than water [[32], [33], [34]].

In fact, there are evidences of environmental pollution caused by over-fertilization in the world [[35], [36], [37], [38]], despite the obvious contribution of fertilizer (organic or mineral) in increasing crops productivity and soil fertility in SSA [14,39,40]. The main constraint of adoption of organic amendment by smallholder farmers are the limited collection capacity and high labor required [41]. Furthermore, most smallholder farmers do not use the recommended dose of mineral fertilizers because of the high cost and limited availability of mineral fertilizers [42]. The limited availability of agricultural resources for smallholder farmers in SSA raises the necessity to focus on the simultaneously improvement of crop productivity through better nutrient- and water and use efficiencies. Otinga et al. [43] demonstrated that localized application of phosphorus fertilizer and farmyard manure increases agronomic efficiency. Ibrahim et al. [44] stated that the combined application of relatively small amount of manure (2000 kg ha^−1^) and mineral fertilizer (kg ha^−1^) was best for achieving high millet yields, nutrient and water use efficiencies in the Sahelian low input millet-based cropping system. These studies suggested that the limited resource use efficiency (like water and nutrient) could be enhanced through nutrient amendment at an appropriate rate, time, place, and from the right source which can optimize roots uptake and reduce potential losses. It is therefore necessary to find optimal combinations of localized manure and fertilizer that will simultaneously improve nutrient- and water-use efficiencies and nutrient balance. Particularly, in the context of hill-placement of manure and fertilizer, the structural relationship among the amount of manure hill-placed with fertilizers, soil properties, grain yield and yield components, aboveground nutrient uptake, nutrient- and water-use efficiencies remain unclear. The present study sets to fill these gaps and to provide a reference for better nutrient management of maize production that also improves our scientific knowledge.

This study therefore aims to evaluate the effect of hill-placed manure and fertilizer on nutrient use efficiency and water use efficiency, using a 4 years (2012–2015) experiment combining hill-placed manure and mineral fertilizer under rainfed conditions in the northern Benin. We hypothesized that the combination of manure and mineral fertilizers at lower rate improves crop growth, nutrient uptake, and nutrient balance; and ultimately enhance nutrient- and water- and use efficiencies. The findings from this research improve our understanding of the additive or synergistic effects of combined hill-placed manure and fertilizer; and the results provide information for a better maize crop production, through the use of farmyard manure and fertilizer in SSA.

## 2 Material and methods

### 2.1 Experimental site

The experiments were carried out at the Agricultural Research Centre of Northern Benin (CRA-Nord) from 2012 till 2015 as described by Tovihoudji et al. [45]. CRA-Nord is located at Ina village (Ina district, municipality of Bembéréké, Borgou department), Northern Benin (9°57′N and 2°42′E, 365 m a.s.l). The climate is tropical sub-humid with a single rainy season occurring between May and October; with an annual rainfall of 1148 mm and an average daily temperature of 27.5 °C (CRA-Nord Climate Database, 1982–2015). The soil is a Lixisol according to the FAO soil classification system. The soil is sandy loam with 5.3% clay in the top 20 cm, acidic with medium phosphorous content and low organic carbon and total nitrogen [45].

### 2.2 Experimental design, installation and management

The experimental plots (4 × 5 m) were arranged in a split-plot design with factorial combinations of FYM (main plot) and mineral fertilizer (sub-plot) in three replicates. FYM was hill-placed at three levels, no manure, 50% and 100% (6 t ha-1) recommended rate, hereafter NM, 3M and 6M respectively (Table 1). FYM used in this study was collected from the barn of the CRA-Nord each year. FYM is a mixture of cattle dung, bedding materials, sand, fodder, and crop residues [46,47]; as commonly used in smallholder farming systems of Africa. The FYM was composed of 14.8 ± 5.6% C, 1.4 ± 0.5% N, 0.3 ± 0.2% P, and 0.9 ± 0.4% K, corresponding to an annual application rates of 885 ± 336 kg C, 84 ± 30 kg N, 18 ± 12 kg P and 54 ± 24 kg K ha^−1^ for the 6M treatment, and half of that for the 3M treatment [45]. Mineral fertilizer was hill-placed, and the three levels were: no fertilizer (NF), 50% (50F) and 100% (100F) of the recommended rate (Table 1). All treatments were maintained in the same plots over the four cropping seasons. Recommended cultivation practices were employed in experimental field preparation for planting (Table 1). Seeds of maize variety DMR-ESR-W (90 days to maturity) were sown on the experimental plots. Due to an experimental bias, data from the 3M treatments are missing in 2014 and 2015. Details of this experiment have been presented in Tovihoudji et al. [45].

**Table 1:** Field management data from 2012 to 2015 at the experimental site.

Growing seasons	Tillage date	Sowing date	Row spacing (cm)	Plant density (plt ha^−1^)	Harvest date	Manure/carbon input (kg ha^−1^)			Fertilizer kg ha^−1^ (N–P–K)		
						NM	3M	6M	NF	50F	100F
2012	24 June	26 June	80	62500	18 Oct	0/0	3000/297	6000/594	0-0-0	38-7-13	76-13-25
2013	25 June	28 June	80	62500	25 Oct	0/0	3000/252	6000/504	0-0-0	38-7-13	76-13-25
2014	30 June	04 July	80	62500	20 Oct	0/0		6000/1464	0-0-0	38-7-13	76-13-25
2015	17 July	20 July	80	62500	05 Nov	0/0		6000/978	0-0-0	38-7-13	76-13-25

DAS: days after sowing, NM: no manure, 3M: 3 t ha^−1^ of applied manure, 6M: 6 t ha^−1^ of applied manure. NF: no fertilizer, 50F: half of recommended fertilizer application rate, 100F: Full recommended fertilizer application rate.

### 2.3 Measurement and calculations

#### 2.3.1 Soil sampling and analysis

At the beginning of the trial (2012), one composite soil sample was taken before sowing, at various randomly selected points from the whole experimental field at 0.2 depth. Furthermore, soil samples were taken just after harvest in 2013, 2014 and 2015 using a soil auger at randomly selected planting hills from the three inner rows of each plot at 0.2 depth. Soil samples were conveyed to the soil and plant analysis laboratory of the International Crop Research Institute for the Semi-Arid Tropics (ICRISAT, Sadore, Niger) and tested for pH (H2O), organic carbon, available P Bray-1, and exchangeable K as highlighted by Tovihoudji et al. [45].

#### 2.3.2 Maize growth and development

Plant leaf area (LA) were recorded in each experimental plot by randomly tagging ﬁve plants from ﬁve diﬀerent planting holes in three middle rows. The green leaf length and width were measured every 15 days with a measuring tape and LA was calculated following Zhang et al. [48] as:

(1) LA=L×W×k

where L is the leaf length, W is the maximum width, and k is a shape factor with the value of 0.75. The leaf area index (LAI) was calculated as the ratio of LA to the horizontal soil surface area occupied by each planting hill [49].

The total dry matter (TDM) was measured by destructive method by cutting a representative whole plant in the border rows of each experimental plots, and samples were initially oven-dried at 105 °C for 30 min after further drying at 70 °C until constant weight and expressed in g m^−2^. Final harvest was manually performed; and grain yields (GY) and biomass yield (BY) were determined from the three inner rows of each experimental pots by cutting each plant at soil level. Samples of grain, cob and stover were oven-dried for moisture content determination and GY and BY were recorded in dry matter basis as described in Tovihoudji et al. [45].

#### 2.3.3 Nutrient uptake, balance and use efficiency

On a yearly basis, a composite sample of FYM was oven-dried at 40 °C, ground, passed through a 1 mm sieve, and analyzed for chemical properties (organic carbon, total N, P and K). Nutrient uptake was determined by randomly sampling three whole plants from two inner rows in each replicate at harvest. The samples were separated into stover (stem, leaves, inﬂorescences, spars and cores) and grains, and oven-dried at 65 °C for 48 h. Sub-samples of the dried plant material were milled for total N, P and K analysis as described in Tovihoudji et al. [45]. All chemical analyses (manure, soils and plants) were carried out at the ICRISAT laboratory (Sadoré, Niger).

A partial nutrient balance metric was used in this study and was calculated by subtracting the quantity of nutrients uptake by the harvested products (grain and crop residue, OUT) from the total quantities of nutrients applied by manure and/or fertilizer (IN) for each plot.

To evaluate the nutrient use efficiencies of maize crop, two indices were calculated: recovery efficiency (NUEre) and internal utilization efficiency (NUEie). NUEre is the quantity of nutrients absorbed per unit of nutrients applied and calculated according to Mohanty et al. [50]:

(2) NUEre=(Nuptake)f−(Nuptake)cQiapplied

NUEie is the ability of maize plant to convert nutrients obtained from all sources (soil, fertilizer, manure) into grain [51] and calculated according to Yan et al. [52]:

(3) NUEie=YieldNutrientuptake

ahere *Qi* is the quantity of nutrient *i* (N, P or K) applied for a treatment. *f* and *c* are the fertilized and control plot respectively.

#### 2.3.4 Soil water storage and water use efficiency

The soil water content (SWC) was measured periodically using a portable soil moisture meter (TRIME-PICO IPH/T3, IMKO Micromodultechnik GmbH). Measurements were taken every 0.1 m from 0 to 0.6 m depth corresponding to the maximum root concentration zone of maize. Before the measurements, the neutron probe of the soil moisture meter was calibrated in-situ for the soil of the experimental ﬁeld using the gravimetric method. Bulk density was determined from undisturbed soil cores. Crop water use per treatment was investigated weekly using the water balance equation [53]:

(4) ETR=I+P−R−D−ΔS

where ETR is evapotranspiration (mm), ΔS is change in soil water content (mm) between two measurements, P is precipitation (mm), I is irrigation (mm), D is drainage and R is runoff. Precipitation data was recorded with a rain gauge installed at the experimental field. Runoff was assumed to be negligible as the experimental ﬁeld was ﬂat. Drainage data were not collected in this study but were extracted from a validated CERES maize model that was calibrated by Tovihoudji et al. [54] with the same experimental data on the same site. Simulated drainage was correlated well with the precipitation (Fig. S1). Drainage amounts was not significantly different between treatments and ranged between 22.73 and 23.99, 10.39–10.81 and 13.86–21.65 and 22.01–22.52 mm in 2012, 2013, 2014 and 2015 respectively.

Seasonal water use in each treatment was calculated as the sum of the weekly water use during entire growing period. Water use efficiency (WUE) was estimated following Zhang et al. [55] as:

(5) WUE=DryyieldEvapotranspiration

### 2.4 Statistical analysis

The statistical analyses were performed using R software package version 4.0.5 (R Foundation for Statistical Computing 2021). The variables were first checked for normal distribution using the Anderson–Darling test, and homogeneity of variance was assessed using Levene's test. ANOVA and the honestly significant difference (HSD)/Tukey's test at an error probability <0.05 were used to evaluate the effect of factors on response variables, and to compare the response variables between factor levels. The relationships between nutrient management strategies, growth, yield variables, water and nutrient use efficiencies was assessed by performing a multiple correlation using *PerformanceAnalytics* package [56] and a structural equation modelling (SEM) using the *lavaan* package [57] in R. Due to high correlation between variables, the N, P and K component of manure, fertilizer, nutrient uptake, NUEre and NUEie were condensed into one representative parameter (dummy variable) by performing a principal component analysis (PCA) before SEM analysis as suggested by Zhang et al. [58]. Overall, the first principal component (PC1) of PCA explained 93.1, 85.2, 88.4 83.1 and 93.3% of the total variation for fertilizer input, manure input, nutrient uptake, RE and IE, respectively.

## 3 Results

### 3.1 Climate condition of the study area

**Fig. 1:**
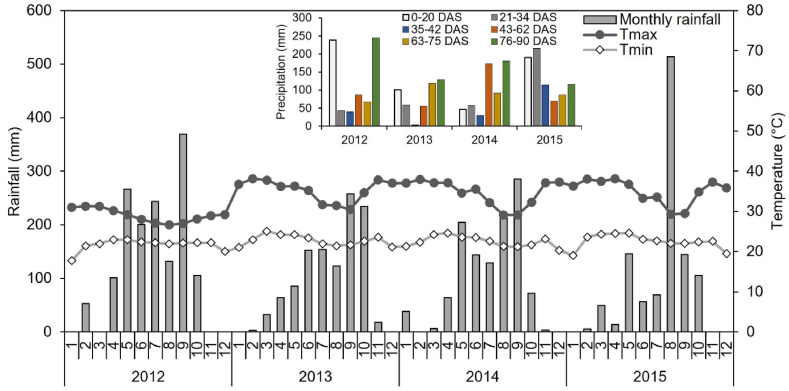
Monthly recorded rainfall and temperature for the four years experiments (2012–2015). DAS: days after sowing, Tmax: maximum temperature, Tmin: minimum temperature. Inner graph represents the cumulative precipitation per maize development stage.0–20 DAS: germination stage, 21–34 DAS: active growth stage, 35–42 DAS: tasseling stage, 43–62 DAS: silking stage, 63–75 DAS: milking stage, 76–90 DAS: physiological maturity.

The amount of total annual rainfall was 1472 mm in 2012, 1126 mm in 2013, 1159 mm in 2014 and 1104 mm in 2015 (Fig. 1); with 875 mm (68% total rain), 761 mm (57%), 659 mm (72%) and 797 mm (72%) from sowing to harvest in 2012, 2013, 2014 and 2015, respectively. The average temperature ranges during the growing seasons were 22.1–27.1 °C, 21.8–31.8 °C, 21.6–30.1 °C and 22.1–30.8 °C in 2012, 2013, 2014 and 2015, respectively (Fig. 1).

### 3.2 Leaf area index and total dry matter

**Fig. 2:**
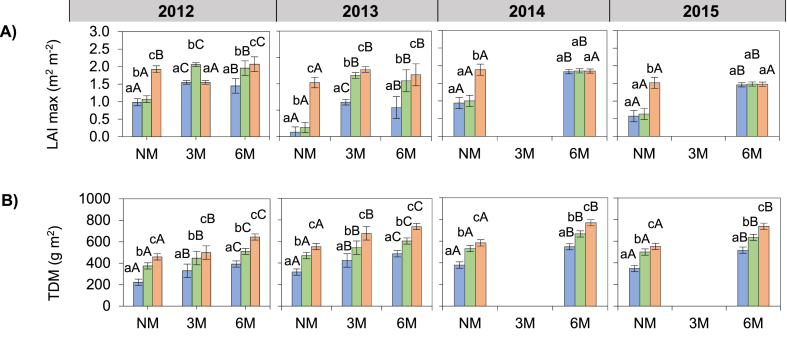
Effect of hill-placed manure and fertilizer on (A) maximum leaf area index and (B) total dry matter at flowering, over four growing seasons (2012–2015). NM: no manure, 3M: 3 t ha-1 of applied manure, 6M: 6 t ha-1 of applied manure. NF: no fertilizer, 50F: half of recommended fertilizer application rate, 100F: Full recommended fertilizer application rate. Error bars represents the standard deviation. Letters “ABC” and “abc” represents mean separation of manure and fertilizer treatments respectively. Data from the 3M treatments are missing in 2014 and 2015.

The leaf area index (LAI) was overall higher at the flowering stage compared to the other stages. It increased from the juvenile stage to the flowering stage, then gradually decreased until the maturity stage (Fig. S2). The maximum LAI (LAI max; at 62 DAS) was highest in 2013 (1.79 m^2^ m^−2^) and 2012 (1.69 m^2^ m^−2^) and lowest in 2014 (1.56 m^2^ m^−2^) and in 2015 (1.19 m^2^ m^−2^) (p < 0.001; Fig. 2A). LAI max was 61 and 44% higher in 3M and 6M, respectively than in NM treatments (p < 0.001; Fig. 2A). LAI max was 18 and 48% higher in 50F and 100F, respectively than in NF treatments (p < 0.001; Fig. 2A). LAI max was 33 and 35% higher in 3M and 6M when combined to 50F, respectively compared to NF treatments (p < 0.001; Fig. 2A). On the contrary, the addition of 100F in 3M and 6M provided only a little additional increase in LAI max compared to 50F treatments. LAI max was significantly improved by 20 and 17% in 50F consecutively compared to NF treatments for 2012 and 2013 while no significant increase was observed in the same treatments in 2014 and 2015 (p < 0.001).

The total dry matter (TDM) was significantly affected by manure and fertilization during the growing period (Fig. S3). TDM at the flowering stage (62 DAS) was higher 2014 (587.7 g m^−2^) and lower in 2012 (440.1 g m^−2^) (p < 0.001; Fig. 2B). TDM was 14 and 37% higher in 3M and 6M, respectively than in NM treatments (p < 0.001; Fig. 2B). TDM was 33% and 59 higher in 50F and 100F, respectively than in NM (p < 0.001; Fig. 2B).

### 3.3 Nutrient uptakes and partial balances

**Fig. 3:**
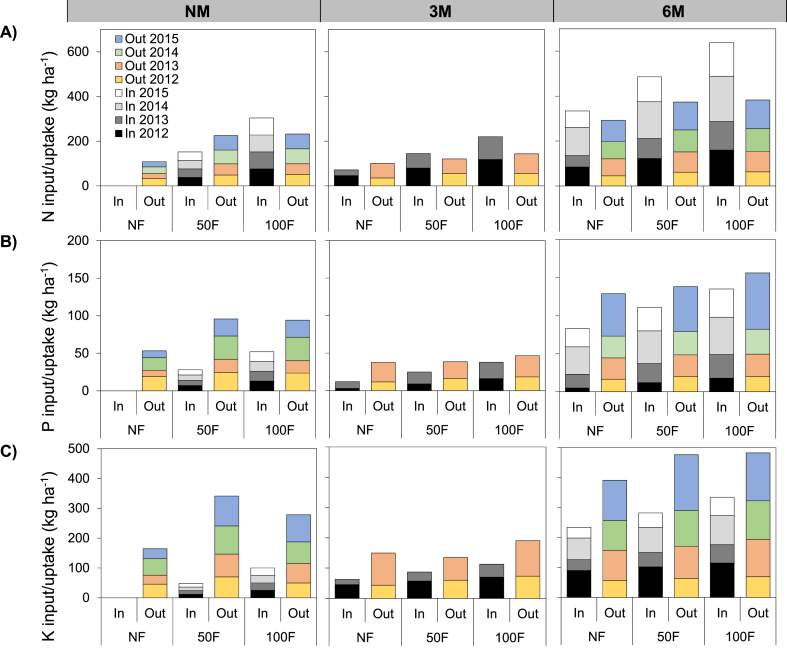
Effect of hill-placed manure and fertilizer on nutrient input, uptake and apparent balances of (A) nitrogen, (B) phosphorous and (C) potassium, over the four growing seasons (2012–2015). NM: no manure, 3M: 3 t ha-1 of applied manure, 6M: 6 t ha-1 of applied manure. NF: no fertilizer, 50F: half of recommended fertilizer application rate, 100F: Full recommended fertilizer application rate. In: Inputs; Out: Uptakes. Data from the 3M treatments are missing in 2014 and 2015.

Nitrogen, P and K uptakes by maize were signiﬁcantly inﬂuenced by nutrient management practices (p < 0.001; Fig. 3A and B, C). Nutrients uptake was higher in 2014 and 2015 than 2012 and 2013 as a result of increasing yields in the fertilized treatments for these years (p < 0.001; Fig. 3A and B, C). N and K uptakes were 29 and 21% respectively higher in 3M than in NM treatments (p < 0.001; Fig. 3A, C); and 86 and 72% respectively higher in 6M than in NM. As for K uptake, only 6M resulted in significant increase (74%) compared to NM treatments (p < 0.001; Fig. 3C). Uptakes of N, P and K were 44, 24 and 21%, respectively higher in 50F than in NF treatments (p < 0.001); and 52, 35 and 72%, respectively higher in 100F than in NF treatments. N uptake was significantly higher under 3M − 100F combination, but not under each combination of NM-100F, and 6M − 100F treatments (p < 0.05; Fig. 3). P uptake was significantly improved in 3M and 6M when combined with 100F but not in NM treatments (p < 0.001; Fig. 3B). Uptakes were highest in 2014 and lowest in 2013 under 3M and 6M treatment except for NM where uptakes were highest in 2012 and lowest in 2015 (p < 0.001; Fig. 3A and B, C). N and P uptakes tended to increase significantly in 50F compared to NF for all years, however addition of 100F led to an additional significant increase only in 2013 and 2014 for N and only in 2013 and 2015 for P, compared to 50F treatments (p < 0.01; Fig. 3A and B).

Partial nutrient balances ranged between −109 and 72, -70 to −42 and −292 to −165 kg ha^−1^ year^−1^, respectively for N, P and K across all fertilization treatments (Fig. 3A and B, C). The combined application of manure and fertilizer generally lowered the extent of negative P and K balances. The application of 3M and 6M led to positive N balance (−33 to 76 and 42–254 kg ha^−1^ year^−1^, for 3M and 6M respectively) compared to NM; and to negative P balance (−26 to −9 and −45 to −20 kg ha^−1^ year^−1^ for 3M and 6M, respectively) and K balance (−87 to −47 and −193 to −149 kg ha^−1^ year^−1^ for 3M and 6M, respectively) (Fig. 3A and B, C).

### 3.4 Recovery efficiency and internal utilization efficiency

**Fig. 4:**
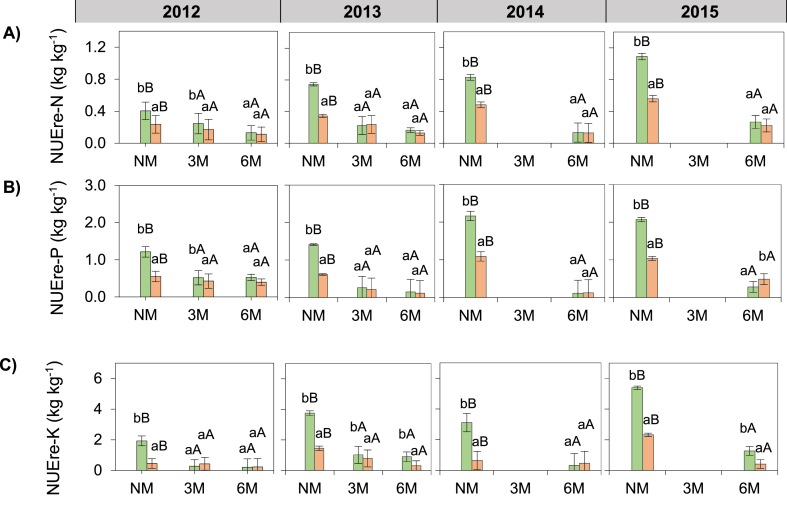
Effect of hill-placed manure and fertilizer on the nutrient recovery efficiencies of maize plant over four growing seasons (2012–2015). NM: no manure, 3M: 3 t ha-1 of applied manure, 6M: 6 t ha-1 of applied manure. NF: no fertilizer, 50F: half of recommended fertilizer application rate, 100F: Full recommended fertilizer application rate. Error bars represents the standard deviation. Letters “ABC” and “abc” represents mean separation of manure and fertilizer treatments, respectively. Data from the 3M treatments are missing in 2014 and 2015.

The TDM basis recovery efficiencies of N and P were in order NUEre_(2015)_ > NUEre_(2013)_ ≥ NUEre_(2014)_ > NUEre_(2012)_ (p < 0.05; Fig. 4A and B); and those of K were NUEre_(2015)_ > NUEre_(2013)_ ≥ NUEre_(2014)_ > NUEre_(2012)_. NUEre-N, NUEre-P and NUEre-K were 63%, 72% and 74% lower in 3M than in NM treatments, respectively (p < 0.001; Fig. 4A and B, C). NUEre-N, NUEre-P and NUEre-K in 6M was similar to the values in 3M treatments (p < 0.001; Fig. 4A and B, C). NUEre-N, NUEre-P and NUEre-K were 38, 42 and 59% respectively, lower in 100F than in 50F treatments (p < 0.01; Fig. 4A and B, C). NUEre-N significantly decreased after fertilizer application in NM, 3M and 6M treatments, while NUEre-P significantly decreased after fertilizer application in NM and 3M but not in 6M treatments. NUEre-K also decreased in response to fertilizer application in NM and 6M but not in 3M treatments. NUEre-N and NUEre-K in 6M were higher in 2013 and lower in 2014 (p < 0.05; Fig. 4A, C) compared to the other years; but NUEre-P was higher in 2012 and lower in 2014 in the 6M treatment.

**Fig. 5:**
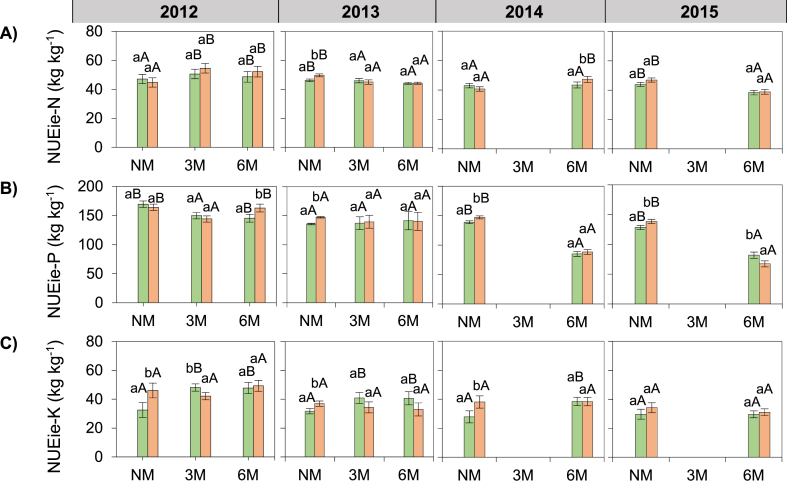
Effect of hill-placed manure and fertilizer on the nutrient internal utilization efficiencies of maize plant over four growing seasons (2012–2015). NM: no manure, 3M: 3 t ha-1 of applied manure, 6M: 6 t ha-1 of applied manure. NF: no fertilizer, 50F: half of recommended fertilizer application rate, 100F: Full recommended fertilizer application rate. Error bars represents the standard deviation. Letters “ABC” and “abc” represents mean separation of manure and fertilizer treatments, respectively. Data from the 3M treatments are missing in 2014 and 2015.

The TDM basis internal utilization efficiencies of N, P and K were in the following order: NUEie_(2012)_ > NUEie_(2013)_ > NUEie_(2014)_ > NUEie_(2015)_ (p < 0.05; Fig. 5A and B, C). NUEie of P was 34 and 12% lower in 3M and 6M, respectively than in NM treatments (p < 0.001; Fig. 5B). NUEie-N and NUEie-P were improved by the addition of 6M in 2012 and 2014 only, compared to NM treatments (p < 0.001; Fig. 5A and B).

### 3.5 Water balance parameters and water use efficiency

The soil water stock was higher in 2015 and lower in 2013, irrespective to measurement dates (p < 0.001, Fig. S4). It was significantly affected by manure at all measurement dates except for 62 DAS, with almost highest values in the manure treatments (Fig. S4).

**Fig. 6:**
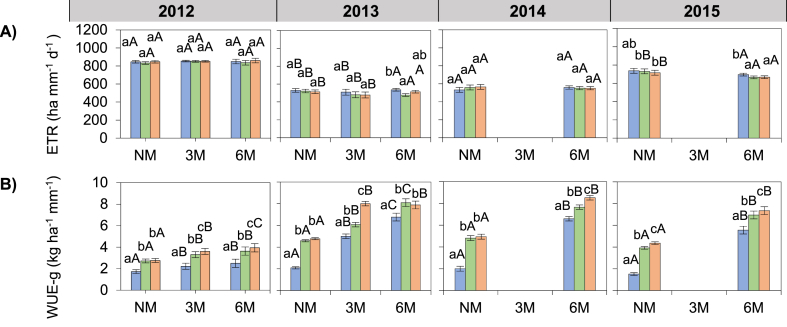
Effect of hill-placed manure and fertilizer on (A) evapotranspiration and (B) water use efficiency of grain yield, over four growing seasons (2012–2015). NM: no manure, 3M: 3 t ha-1 of applied manure, 6M: 6 t ha-1 of applied manure. NF: no fertilizer, 50F: half of recommended fertilizer application rate, 100F: Full recommended fertilizer application rate. Error bars represents the standard deviation. Letters “ABC” and “abc” represents mean separation of manure and fertilizer treatments respectively. Data from the 3M treatments are missing in 2014 and 2015.

The evapotranspiration was higher in 2012 (847.8 mm) and lower in 2013 (507.2 mm) (p < 0.001; Fig. 6A). ETR was 2% lower in 6M than in NM treatments (p < 0.001; Fig. 6A); and was reduced by 2 and 7% following the application of 6M compared to NM in 2013 and 2015, while the same 6M provided a significant change in ETR compared to NM in 2012 and 2014 (p < 0.01; Fig. 6A).

The water use efficiency in grain yield (WUE-g) was higher in 2013 (5.92 kg ha^−1^ mm^−1^) and 2014 (5.75 kg ha^−1^ mm^−1^) than 2015 (4.94 kg ha^−1^ mm^−1^) and 2012 (2.94 kg ha^−1^ mm^−1^) (p < 0.001; Fig. 6B). WUE-g was 40 and 88% higher for 3M and 6M, respectively than in NM treatments (p < 0.01; Fig. 6B). Within fertilized treatments, WUE-g was 26 and 45% higher for 50F and 100F, respectively than in NF treatments (p < 0.01; Fig. 6B). WUE-g was increased by 129% after the application of 100F in the NM treatment, but, adding 100F in 3M and 6M treatments resulted in a 61 and 39% increase in WUE-g compared to NF (p < 0.01; Fig. 6B). WUE-g increased by 40% following the application of 6M compared to NM in 2012, while the same 6M provided a greater improvement in WUE-g compared to NM in 2013 (99%), 2014 (93%) and 2015 (103%) (p < 0.01; Fig. 6B). WUE-g significantly increased with the increasing fertilizer rate in 2013 and 2014, except in 2012 and 2014 where 50F and 100F improved WUE-g to an equal extent compared to NF treatments (p < 0.01; Fig. 6B). The water use efficiency of biomass yield (WUE-b) showed similar pattern with WUE-g (data not shown).

### 3.6 Relationships among growth, yield parameters, and nutrient- and water-use efficiencies

**Fig. 7:**
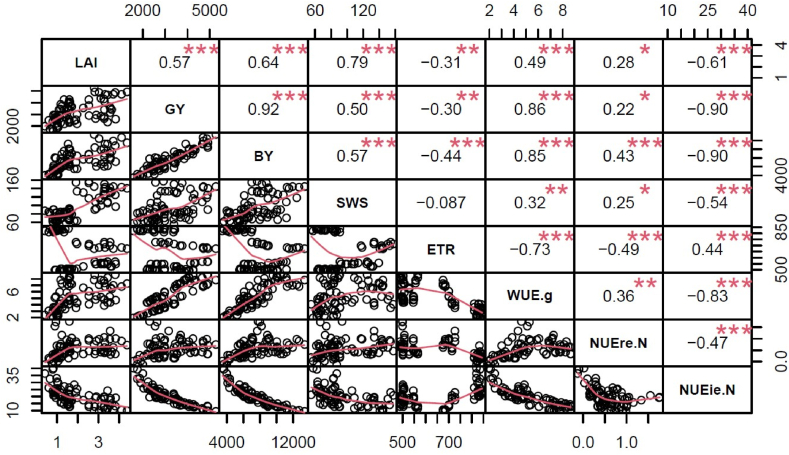
Correlation plot showing the relationship between growth, yield and environmental variables on WUE and NUEs. The numbers represent the correlation coefficients while the “*” represent the significance (* = <0.05, ** =<0.01, *** = <0.001). LAI: maximum leaf area index, GY: grain yield, BY: total biomass yield at harvest, SWS: soil water stock at flowering stage, ETR: evapotranspiration, WUE.g: water use efficiency of grain yield, NUEreN: nutrient recovery efficiency of N, NUEieN: nutrient internal utilization efficiency of N.

Significant positive correlations occurred between WUE-g and LAI, grain yield, biomass yield, soil water stock and NUEre-N, with coefficient values ranging between 0.32 and 0.86 (Fig. 7). On the contrary, WUE-g was negatively correlated with NUEie-N (r = −0.83; Fig. 7). NUEre-N was positively correlated with LAI, grain yield, biomass yield and soil water stock, with coefficient values ranging from 0.22 to 0.43 (Fig. 7). However, NUEre-N was negatively correlated with ETR (r = −0.49; Fig. 7). Negative correlations were found between the NUEie-N and LAI, grain yield, biomass yield and soil water stock, with values ranging from −0.47 to −0.90 (Fig. 7). However, NUEie-N was positively correlated with ETR (r = 0.44; Fig. 7).

**Fig. 8:**
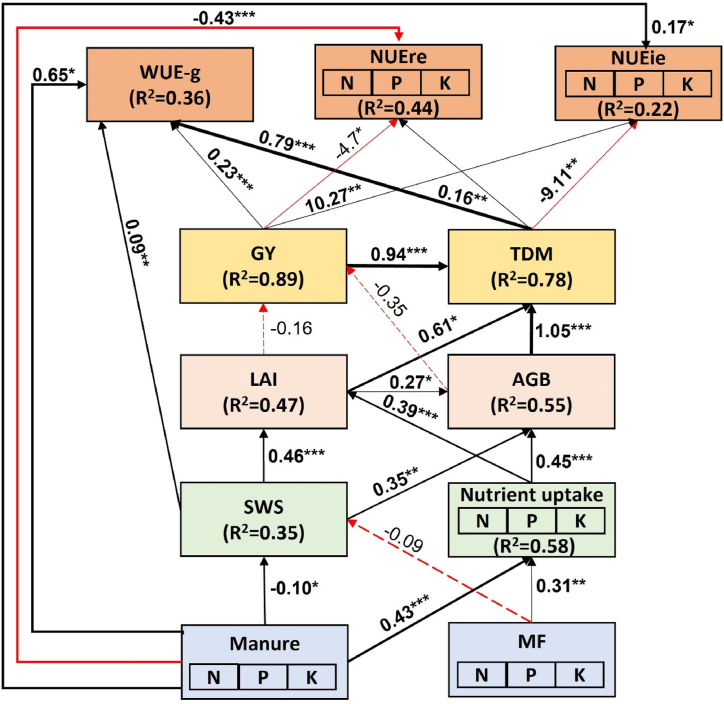
Structural equation model showing the relationship between nutrient management strategies, growth, yield, water and nutrient use efficiencies. Chi-square (χ2) = 232.35; p-value = 0.03; Degree of freedom (df) = 33; RMSEA = 0.31; Goodness of fit (GFI) = 0.98; Comparative fit (CFI) = 0.86. The black and red color of the arrow represents positive and negative paths respectively. Only significant relationships are shown. Symbol *, ** and *** represents the level of significance at 0.05, 0.01 and 0.001 respectively for the path coefficient. Number on the arrow represent the path coefficient. Arrow width represent the strength of the path coefficient. MF: mineral fertilizer, SWS: soil water storage, LAI: maximum leaf area index, TDM: total dry matter at flowering, GY: grain yield, BY: biomass yield, WUE-g: water use efficiency of grain, NUEre: nutrient recovery efficiency, NUEie: nutrient internal utilization efficiency. (For interpretation of the references to color in this figure legend, the reader is referred to the Web version of this article.)

Structural equation model reveals significant direct effects of manure application on nutrient uptake (λ = 0.43; p < 0.001), soil water stock (λ = −0.10; p < 0.05), WUE-g (λ = 0.65; p < 0.05), NUEre (λ = −0.43; p < 0.001) and NUEie (λ = 0.17; p < 0.05) (Fig. 8). Nutrient management (manure and fertilizer) influenced growth (LAI and TDM) via nutrient uptake. Grain yield positively influenced WUE-g (λ = 0.23; p < 0.001) and NUEie (λ = 10.27; p < 0.01), but negatively NUEre (λ = −4.7; p < 0.05). Biomass yield positively influenced WUE-g (λ = 0.79; p < 0.001) and NUEre (λ = 0.16; p < 0.01), but negatively influenced NUEie (λ = −9.11; p < 0.01). Overall, the SEM explained 36%, 44% and 22% of the variance in WUE-g, NUEre and NUEie, respectively (Fig. 8).

## 4 Discussion

### 4.1 Nutrient uptake, balance and use efficiencies as affected by hill-placed manure and fertilizer application

Our findings show that manure and mineral fertilizer amendment improved maize crops nutrient uptakes, with no significant difference between 50F and 100F under mineral fertilizer supply. Nutrient uptake was generally higher under treatment with combined application of manure and fertilizer compared to sole manure or fertilizer application. In fact, nutrient application from organic and inorganic sources leads to better nutrients availability and further enhanced the nutrient content in the plants’ stover and grain [59]. Muazu and Uyovbisere [60] also reported that manure can boost nutrient uptake; and previous have highlighted that the combined application of nutrients from organic and inorganic source improves N, P and K uptake [61,62].

Nutrient uptake exceeding input rate led to negative partial balance mainly for NF and 50F under NM, while it was positive under 6M across all fertilizer treatments (Fig. 3). The positive N partial balance in the 6M treatments can be attributed to the cumulative effect of manure and fertilizer on the soil enrichment, which ensure a higher quantity of available N in the soil even after plants uptake [63]. On the contrary, the negative nutrient balance under sole fertilizer application treatment suggests that plant could extract the maximum amount of available N in the soil thereby leaving the soil nutrient status poor. These findings corroborate with those of Adamtey et al. [64] who also recorded a negative partial balance in conventional system and a positive partial balance in an organic system of a maize-based cropping system in Kenya. Therefore, the application of mineral fertilizer alone in maize cropping is unsustainable since the soil nutrient status could lower after cropping, while the combination of manure with fertilizer ensures a replenishment of the soil nutrient pool thereby maintaining soil fertility.

The NUEre and NUEie help to better understand the factors influencing amendment efficiency [65]. Here, increasing the fertilizer rate decreased NUEre, and increasing the rate of farmyard manure stabilized the NUEre. That is the increasing rate of fertilizer might not necessarily produce a higher nutrient use efficiency since highest nutrient use efficiency occurred under low amendment input. The combination of manure and fertilizer can be beneficial due to the immobilization of nutrient by the manure coupled with the readily available nutrients in the mineral fertilizer [59,66]. This result is in line with Tofa et al. [67] who reported that highest nutrient recovery efficiency was obtained when the lowest N and P was applied. Oppositely, Krishnaprabu [68] reported that the combination of farmyard manure and fertilizer boosted nutrient recovery by more than 100% compared to fertilizer alone. In fact, the nutrient uptake was higher under farmyard manure and fertilizer combination than the amount of nutrient input. The response of NUEre suggest that maize better responded to mineral fertilization than integrated nutrient fertilization, since mineral fertilizers more rapidly supply available nutrients to plants [69].

The NUEie was enhanced by manure application and the addition of fertilizer. Although not significant, the combination of manure and fertilizer increased the NUEie more than fertilizer or manure alone. However, the NUEie in 50F and 100F treatments across all manure strata were mostly similar. The higher NUEie resulting from the combination of manure and fertilizer may be due to the higher yield obtained from such treatment. Our results are in contradiction with Fazily et al. [70] who reported that mineral fertilization results in the highest NUEie, which might be due to obtained high yield under combination of manure and fertilizer. Since NUEie is the additional yield obtained per unit of nutrient uptake [71], any strategy that enhances biomass production will consequently improve the NUEie.

### 4.2 Water use efficiency as affected by hill-placed manure and fertilizer application

The water use efficiency significantly increased with increasing manure application rate (Fig. 6). The improvement of WUE following manure application can be attributed to beneficial effect of manure on soil physical properties such as bulk density, which promotes effective water transport to plant root zone [72]. The enhancement of WUE following the hill-placement application of manure has been reported by Ibrahim et al. [44]. In this study, manure had greater effect on WUE in 2013, 2014 and 2015 compared to 2012; due to the higher yield in these years that was attributed to the residual effect of manure compared to 2012. Similarly, Tahir et al. [73] also reported the positive residual effect of manure on WUE. Manure application has a great residual potential in long term improvement of soil physical properties, making its application a sustainable nutrient management option [74,75]. The WUE also increased after fertilizer application, but 50F and 100F responded similarly to WUE improvement compared to NM treatment. Fertilizer was beneficial to WUE probably because of higher yield resulting from fertilizer application in this study. Furthermore, fertilizer supply in soils promotes crop and root growth, thereby roots can penetrate deeper soil layer for efficient water uptake [76,77].

### 4.3 Structural relationship between manure, fertilizer, growth, yield, water- and nutrient-use efficiencies

The nutrient recovery efficiency (NUEre) had a direct and negative relationship with manure application according to the SEM (Fig. 8). This negative relationship may be due to the low NPK content in farmyard manure. Mohamed et al. [78] reported similar reduction in crop recovery efficiency after the application of manure. However, the SEM results also suggested that application of manure or fertilizer indirectly improved NUEre by directly improving nutrient uptake and total dry matter at harvest (Fig. 8). The improvement of nutrient uptake may be due to the role of manure in increasing soil microbial activities which in turns reduces the bulk density of the soil and promote better soil nutrient retention [59]. Previous studies reported that TDM can be improved by the alteration of soil physico-chemical properties resulting from the addition of organic manure [79,80]. The improved soil physico-chemical properties could be attributed to the increase in soil organic matter and nutrient availability which promotes plant growth as suggested by Seleiman and Abdalaal [81]. Peralta-Antonio et al. [82] also reported an increase in recovery efficiency related to an increase in nutrient uptake and TDM. The internal utilization efficiency (NUEie) had a direct and positive relationship with manure application. Nonetheless, the SEM results also suggest that application of manure and/or fertilizer indirectly reduced NUEie by directly improving nutrient uptake and total dry matter at harvest (Fig. 8); as an increased in TDM was associated with a decrease in NUEie. This can be explained by the enhancement of nutrient uptake and yield resulting from the application of manure. According to previous results, NUEie increases following the application of organic amendment [83,84], while it decreases with the increasing nutrient uptake [85].

The water use efficiency of grain yield (WUE-g) had a direct and positive relationship with manure application. Similarly, manure application also indirectly affected the WUE-g, through the improvement of soil water content (Fig. 8). Manure can increase soil macro-porosity which promotes better water storage [86,87]. The improvement in WUE due to an increase in soil water is in contradiction with Ullah et al. [9] who stated that less water can increase WUE due to a decrease in soil evaporation. Indeed, the high soil water improved LAI and biomass which in turn contributed to better grain yield and consequently better WUE. Finally, nutrient management (manure and/or fertilizer) had an indirect effect on WUE-g by improving nutrient uptake, yield and total dry matter (Fig. 8), due to the interactive effect of manure and fertilizer on soil nutrient replenishment [88].

### 4.4 Implication for smallholder farmers in rainfed agriculture

Smallholder farmers can achieve higher nutrient- and water-use productivities through the beneficial effect of combined hill-placed mineral fertilizer and farmyard manure to intensify maize crop production. In fact, most farmers already use half of recommended fertilizer rate due to financial limitation and limited availability of chemical fertilizer [89], but the mineral fertilizers are often applied with no manure combination [45]. While using 50% of fertilizer rate alone is beneficial for nutrient recovery efficiency maximization, its combination with manure can lead to even better results when considering internal utilization efficiency and water use efficiency. Limited collection capacity of farmyard manure and high labor are limiting factors encountered by smallholder farmers to manure adoption [90]. This is due to the high manure quantity recommended by extension services (6–10 t ha^−1^). This study, however, demonstrates that the combination of manure, even in small quantity could be beneficial as it simultaneously improved nutrient- and water-use productivities. Indeed, the application of 3 t ha^−1^ combined with 50% of fertilizer recommended rate appeared as the option that maximized nutrient use efficiency. However, for a higher water use efficiency, smallholder farmers who have collection capacity can increase the manure rate to 6 t ha^−1^ to concurrently lead to higher yield. The integrated crop-livestock management can be encouraged to increase the manure collection capacity of smallholder farmers in the region because of the availability of cattle herds. Ahozonlin and Dossa [91] reported that the adoption of integrated crop-livestock farming in Benin promotes the use of crop residues as feed for animals and recycling feaces as manure; thus reducing the negative impact of the loss of natural grazing lands on cattle herds by providing supplemental feed for animals.

Further research could investigate the feasibility and economic efficiency of an integrated crop-livestock tailored to local realities in the study region. As for mineral fertilizers, an alternative to reduce nutrient leaching caused by high rate of mineral fertilizer could be to split the application of fertilizer across the crop growing stage. Similarly, Deng et al. [92] reported that maize yield increased by 6.7–11.5% with four split applications of N compared to single application. The same authors highlighted that N split application leads to higher N concentration in plant and higher N use efficiency. Thus, further studies are required in SSA to validate this strategy for improving N application in the right amount, time, place and source, as recommended in the context of precision agriculture.

## 5 Conclusion

This study suggests that the combination of hill-placement of farmyard manure and synthetic fertilizer is an effective strategy to improve nutrient- and water-use efficiencies of maize crops. The results show that increasing the rate of manure and/or fertilizer led to a decrease in nutrient recovery efficiency, but the decrease rate was lower under combined application, while internal utilization efficiency increased with increasing manure and fertilizer amounts. Furthermore, the water use efficiency was higher under combined application of manure and synthetic fertilizer. These findings provide practical recommendations for farmers in Benin to optimize maize production while minimizing the negative environmental impacts of fertilizer use. Combining 3000 kg ha^−1^ of farmyard manure with half of fertilizer recommended rate (100 kg ha^−1^) could be suggested as an optimal nutrient management practice for water and-nutrient use efficiencies, and subsequently maize production in the region. The practical recommendation of this study could be more robust if famers were associated in the experimental setup and management. Future studies should target the other agro-ecological zones in Benin, and also consider other widely cultivated crops in the study area to reduce the yield gaps and promote food security in the study region.

## Author contribution statement

Mouiz W. I. A. Yessoufou: Analyzed and interpreted the data and Wrote the paper.

Pierre G. Tovihoudji: Conceived and designed the experiments; Performed the experiments and Wrote the paper.

Zakari Sissou: Analyzed and interpreted the data and Wrote the paper.

André Adjogboto: Performed the experiments and Wrote the paper.

A. Jonas Djenontin and P. B. Irénikatché Akponikpè: Conceived and designed the experiments and Wrote the paper.

## Data availability statement

Data used in this study will be used for modeling, so the authors do not want to share the data now.

## Funding

This study was funded by Climate Change Agriculture and Food Security (CCAFS) program of CGIAR and the West Africa Agricultural Productivity Program (WAAPP-Benin).

## Declaration of competing interest

The authors declare that they have no known competing financial interests or personal relationships that could have appeared to influence the work reported in this paper.

## References

[1] Afreh D., Zhang J., Guan D., Liu K., Song Z., Zheng C., Deng A., Feng X., Zhang X., Wu Y. (2018). Long-term fertilization on nitrogen use efficiency and greenhouse gas emissions in a double maize cropping system in subtropical China. Soil Tillage Res..

[2] Tully K., Sullivan C., Weil R., Sanchez P. (2015). The state of soil degradation in Sub-Saharan Africa: baselines, trajectories, and solutions. Sustainability.

[3] Monaghan J.M., Daccache A., Vickers L.H., Hess T.M., Weatherhead E.K., Grove I.G., Knox J.W. (2013). More ‘crop per drop’: constraints and opportunities for precision irrigation in European agriculture. J. Sci. Food Agric..

[4] Sangare S.K., Compaore E., Buerkert A., Vanclooster M., Sedogo M.P., Bielders C.L. (2012). Field-scale analysis of water and nutrient use efficiency for vegetable production in a West African urban agricultural system. Nutrient Cycl. Agroecosyst..

[5] Liang G., Sun P., Waring B.G. (2022). Nitrogen agronomic efficiency under nitrogen fertilization does not change over time in the long term: evidence from 477 global studies. Soil Tillage Res..

[6] Koech R., Langat P. (2018). Improving irrigation water use efficiency: a review of advances, challenges and opportunities in the Australian context. Water.

[7] Brunel-Saldias N., Seguel O., Ovalle C., Acevedo E., Martínez I. (2018). Tillage effects on the soil water balance and the use of water by oats and wheat in a Mediterranean climate. Soil Tillage Res..

[8] Hatfield J.L., Dold C. (2019). Water-use efficiency: advances and challenges in a changing climate. Front. Plant Sci..

[9] Ullah H., Santiago-Arenas R., Ferdous Z., Attia A., Datta A. (2019). Improving water use efficiency, nitrogen use efficiency, and radiation use efficiency in field crops under drought stress: a review. Adv. Agron..

[10] Mvena Z.S.K. (2016). Climate Change and Multi-Dimensional Sustainability in African Agriculture.

[11] Saidia P.S., Asch F., Kimaro A.A., Germer J., Kahimba F.C., Graef F., Semoka J.M.R., Rweyemamu C.L. (2019). Soil moisture management and fertilizer micro-dosing on yield and land utilization efficiency of inter-cropping maize-pigeon-pea in sub humid Tanzania. Agric. Water Manag..

[12] Andriamananjara A., Rakotoson T., Razafimbelo T., Rabeharisoa L., Razafimanantsoa M.P., Masse D. (2019). Farmyard manure improves phosphorus use efficiency in weathered P deficient soil. Nutrient Cycl. Agroecosyst..

[13] Dhaliwal S.S., Sharma V., Shukla A.K., Verma V., Kaur M., Singh P., Gaber A., Hossain A. (2023). Effect of addition of organic manures on basmati yield, nutrient content and soil fertility status in north-western India. Heliyon.

[14] Tovihoudji P.G., Akponikpè P.B.I., Agbossou E.K., Bertin P., Bielders C.L. (2017). Fertilizer microdosing enhances maize yields but may exacerbate nutrient mining in maize cropping systems in northern Benin. Field Crop. Res..

[15] Likpètè D.D., Adjogboto A., Akponikpè P.B.I., Djènontin A.J., Baco M.N., Sossa-Vihotogbé C.N.A. (2021). Fertilizer microdosing enhances biomass yield and water use efficiency of African indigenous leafy vegetable. Int. J. Veg. Sci..

[16] Nayak H.S., Parihar C.M., Mandal B.N., Patra K., Jat S.L., Singh R., Singh V.K., Jat M.L., Garnaik S., Nayak J., Abdallah A.M. (2022). Point placement of late vegetative stage nitrogen splits increase the productivity, N-use efficiency and profitability of tropical maize under decade long conservation agriculture. Eur. J. Agron..

[17] Tang J., Su L., Fang Y., Wang C., Meng L., Wang J., Zhang J., Xu W. (2023). Moderate nitrogen reduction increases nitrogen use efficiency and positively affects microbial communities in agricultural soils. Agriculture.

[18] Descheemaeker K., Amede T., Haileslassie A. (2010). Improving water productivity in mixed crop–livestock farming systems of sub-Saharan Africa. Agric. Water Manag..

[19] Esilaba A.O., Njiru E., Ruto R., Omondi S.P., Kwena K.M., Thuranira E.G., Mwangi J.A., Simiyu A.W. (2020).

[20] Masikati P. (2011).

[21] Belay S.A., Assefa T.T., Prasad P.V., Schmitter P., Worqlul A.W., Steenhuis T.S., Reyes M.R., Tilahun S.A. (2020). The response of water and nutrient dynamics and of crop yield to conservation agriculture in the Ethiopian highlands. Sustainability.

[22] Zhang K., Li Y., Wei H., Zhang L., Li F.-M., Zhang F. (2022). Conservation tillage or plastic film mulching? A comprehensive global meta-analysis based on maize yield and nitrogen use efficiency. Sci. Total Environ..

[23] Zhang Q., Wang S., Sun Y., Zhang Y., Li H., Liu P., Wang X., Wang R., Li J. (2022). Conservation tillage improves soil water storage, spring maize (Zea mays L.) yield and WUE in two types of seasonal rainfall distributions. Soil Tillage Res..

[24] Bihari B., Singh Y.K., Shambhavi S., Mandal J., Kumar S., Kumar R. (2022). Nutrient use efficiency indices of N, P, and K under rice-wheat cropping system in LTFE after 34th crop cycle. J. Plant Nutr..

[25] Darjee S., Shrivastava M., Langyan S., Singh G., Pandey R., Sharma A., Khandelwal A., Singh R. (2022).

[26] Zhai L., Wang Z., Zhai Y., Zhang L., Zheng M., Yao H., Lv L., Shen H., Zhang J., Yao Y., Jia X. (2022). Partial substitution of chemical fertilizer by organic fertilizer benefits grain yield, water use efficiency, and economic return of summer maize. Soil Tillage Res..

[27] Kihara J., Bolo P., Kinyua M., Nyawira S.S., Sommer R. (2020). Soil health and ecosystem services: lessons from sub-Sahara Africa (SSA). Geoderma.

[28] Sileshi G.W., Jama B., Vanlauwe Be, Negassa W., Harawa R., Kiwia A., Kimani D. (2019). Nutrient use efficiency and crop yield response to the combined application of cattle manure and inorganic fertilizer in sub-Saharan Africa. Nutrient Cycl. Agroecosyst..

[29] Vanlauwe B., Bationo A., Chianu J., Giller K.E., Merckx R., Mokwunye U., Ohiokpehai O., Pypers P., Tabo R., Shepherd K.D. (2010). Integrated soil fertility management: operational definition and consequences for implementation and dissemination. Outlook Agric..

[30] Mutegi J., Ameru J., Harawa R., Kiwia A., Njue A. (2018). Soil health and climate change: implications for food security in Sub-Saharan Africa. Int. J. Dev. Sustain..

[31] Salama H.S.A., Nawar A.I., Khalil H.E., Shaalan A.M. (2021). Improvement of maize productivity and N use efficiency in a No-tillage irrigated farming system: effect of cropping sequence and fertilization management. Plants.

[32] Srivastava A.K., Mboh C.M., Gaiser T., Ewert F. (2017). Impact of climatic variables on the spatial and temporal variability of crop yield and biomass gap in Sub-Saharan Africa-a case study in Central Ghana. Field Crop. Res..

[33] Tittonell P., Giller K.E. (2013). When yield gaps are poverty traps: the paradigm of ecological intensification in African smallholder agriculture. Field Crop. Res..

[34] Twomlow S., Rohrbach D., Dimes J., Rusike J., Mupangwa W., Ncube B., Hove L., Moyo M., Mashingaidze N., Mahposa P. (2011). Innovations as Key to the Green Revolution in Africa.

[35] Dou X., Wang R., Zhou X., Gao F., Yu Y., Li C., Zheng C. (2022). Soil water, nutrient distribution and use efficiencies under different water and fertilizer coupling in an apple–maize alley cropping system in the Loess Plateau, China. Soil Tillage Res..

[36] Shi N., Zhang Y., Li Y., Luo J., Gao X., Jing Y., Bo L. (2018). Water pollution risk from nitrate migration in the soil profile as affected by fertilization in a wheat-maize rotation system. Agric. Water Manag..

[37] Song H., Che Z., Cao W., Huang T., Wang J., Dong Z. (2016). Changing roles of ammonia-oxidizing bacteria and archaea in a continuously acidifying soil caused by over-fertilization with nitrogen. Environ. Sci. Pollut. Control Ser..

[38] Song Q., Fu H., Shi Q., Shan X., Wang Z., Sun Z., Li T. (2022). Overfertilization reduces tomato yield under long-term continuous cropping system via regulation of soil microbial community composition. Front. Microbiol..

[39] Mathenge C., Thuita M., Masso C., Gweyi-Onyango J., Vanlauwe B. (2019). Variability of soybean response to rhizobia inoculant, vermicompost, and a legume-specific fertilizer blend in Siaya County of Kenya. Soil Tillage Res..

[40] Nafi E., Webber H., Danso I., Naab J.B., Frei M., Gaiser T. (2020). Interactive effects of conservation tillage, residue management, and nitrogen fertilizer application on soil properties under maize-cotton rotation system on highly weathered soils of West Africa. Soil Tillage Res..

[41] Ndambi O.A., Pelster D.E., Owino J.O., De Buisonje F., Vellinga T. (2019). Manure management practices and policies in sub-Saharan Africa: implications on manure quality as a fertilizer. Front. Sustain. Food Syst..

[42] Ariga J., Mabaya E., Waithaka M., Wanzala-Mlobela M. (2019). Can improved agricultural technologies spur a green revolution in Africa? A multicountry analysis of seed and fertilizer delivery systems. Agric. Econ..

[43] Otinga A.N., Pypers P., Okalebo J.R., Njoroge R., Emong’ole M., Six L., Vanlauwe B., Merckx R. (2013). Partial substitution of phosphorus fertiliser by farmyard manure and its localised application increases agronomic efficiency and profitability of maize production. Field Crop. Res..

[44] Ibrahim A., Abaidoo R.C., Fatondji D., Opoku A. (2015). Hill placement of manure and fertilizer micro-dosing improves yield and water use efficiency in the Sahelian low input millet-based cropping system. Field Crop. Res..

[45] Tovihoudji P.G., Akponikpè P.B.I., Adjogboto A., Djenontin J.A., Agbossou E.K., Bielders C.L. (2018). Combining hill-placed manure and mineral fertilizer enhances maize productivity and profitability in northern Benin. Nutrient Cycl. Agroecosyst..

[46] Hoffmann I., Gerling D., Kyiogwom U.B., Mané-Bielfeldt A. (2001). Farmers' management strategies to maintain soil fertility in a remote area in northwest Nigeria. Agric. Ecosyst. Environ..

[47] Rinasoa S., Rakotoson T., Rabeharisoa L., Tsujimoto Y., Nishigaki T. (2023). Farmyard manure application increases lowland rice yield in phosphorus-deficient soils, but not in soils with high pH and phosphorus-fixing capacity. Field Crop. Res..

[48] Zhang W., Chen X.X., Liu Y.M., Liu D.Y., Du Y.F., Chen X.P., Zou C.Q. (2018). The role of phosphorus supply in maximizing the leaf area, photosynthetic rate, coordinated to grain yield of summer maize. Field Crop. Res..

[49] Wang H., Yu Z., Shi Y., Zhang Y. (2020). Effects of tillage practices on grain yield formation of wheat and the physiological mechanism in rainfed areas. Soil Tillage Res..

[50] Mohanty S., Nayak A.K., Swain C.K., Dhal B.R., Kumar A., Kumar U., Tripathi R., Shahid M., Behera K.K. (2020). Impact of integrated nutrient management options on GHG emission, N loss and N use efficiency of low land rice. Soil Tillage Res..

[51] Dobermann A. (2007). Fertilizer Best Management Practices: General Principles, Strategy for Their Adoption and Voluntary Initiatives vs Regulations.

[52] Yan S., Wu Y., Fan J., Zhang F., Guo J., Zheng J., Wu L., Lu J. (2022). Quantifying nutrient stoichiometry and radiation use efficiency of two maize cultivars under various water and fertilizer management practices in northwest China. Agric. Water Manag..

[53] Cao Q., Wang Z., Yang X., Shen Y. (2021). The effects of cocksfoot cover crop on soil water balance, evapotranspiration partitioning, and system production in an apple orchard on the Loess Plateau of China. Soil Tillage Res..

[54] Tovihoudji P.G., Akponikpè P.B.I., Agbossou E.K., Bielders C.L. (2019). Using the DSSAT model to support decision making regarding fertilizer microdosing for maize production in the sub-humid region of Benin. Front. Environ. Sci..

[55] Zhang X., Dong Z., Wu X., Gan Y., Chen X., Xia H., Kamran M., Jia Z., Han Q., Shayakhmetova A. (2021). Matching fertilization with water availability enhances maize productivity and water use efficiency in a semi-arid area: mechanisms and solutions. Soil Tillage Res..

[56] Peterson B.G., Carl P., Boudt K., Bennett R., Ulrich J., Zivot E., Cornilly D., Hung E., Lestel M., Balkissoon K. (2018). Package ‘performanceanalytics. ’ R Team Cooperation..

[57] Rosseel Y. (2012). lavaan: an R package for structural equation modeling. J. Stat. Software.

[58] Zhang Q., Song Y., Wu Z., Yan X., Gunina A., Kuzyakov Y., Xiong Z. (2020). Effects of six-year biochar amendment on soil aggregation, crop growth, and nitrogen and phosphorus use efficiencies in a rice-wheat rotation. J. Clean. Prod..

[59] Phares C.A., Amoakwah E., Danquah A., Akaba S., Frimpong K.A., Mensah T.A. (2022). Improved soil physicochemical, biological properties and net income following the application of inorganic NPK fertilizer and biochar for maize production. Acta Ecol. Sin..

[60] Muazu H., Uyovbisere E.O. (2022). Nitrogen uptake in cow dung and poultry manure treated maize (Zea mays L.) under different irrigation intervals in Sudan Savanna Ecology, Sokoto, Nigeria. Journal of Agriculture and Environment.

[61] Ngala A.L., Digmari F.U., Ndirmbula J.B., Abdullahi R. (2021). Effects of NPK, bio-fertilizers and manures on growth of maize (Zea mays L.) and soil nutrients content in maiduguri, Nigeria. European Journal of Agriculture and Food Sciences.

[62] Qaswar M., Jing H., Ahmed W., Dongchu L., Shujun L., Lu Z., Cai A., Lisheng L., Yongmei X., Jusheng G., Huimin Z. (2020). Yield sustainability, soil organic carbon sequestration and nutrients balance under long-term combined application of manure and inorganic fertilizers in acidic paddy soil. Soil Tillage Res..

[63] Midya A., Saren B.K., Dey J.K., Maitra S., Praharaj S., Gaikwad D.J., Gaber A., Alhomrani M., Hossain A. (2021). Crop establishment methods and integrated nutrient management improve: Part ii. nutrient uptake and use efficiency and soil health in rice (Oryza sativa L.) field in the lower indo-gangetic plain, India. Agronomy.

[64] Adamtey N., Musyoka M.W., Zundel C., Cobo J.G., Karanja E., Fiaboe K.K.M., Muriuki A., Mucheru-Muna M., Vanlauwe B., Berset E., Messmer M.M., Gattinger A., Bhullar G.S., Cadisch G., Fliessbach A., Mäder P., Niggli U., Foster D. (2016). Productivity, profitability and partial nutrient balance in maize-based conventional and organic farming systems in Kenya. Agric. Ecosyst. Environ..

[65] Melino V.J., Tester M.A., Okamoto M. (2022). Strategies for engineering improved nitrogen use efficiency in crop plants via redistribution and recycling of organic nitrogen. Curr. Opin. Biotechnol..

[66] Dimkpa C., Adzawla W., Pandey R., Atakora W.K., Kouame A.K., Jemo M., Bindraban P.S. (2023). Fertilizers for food and nutrition security in sub-Saharan Africa: an overview of soil health implications. Frontiers in Soil Science.

[67] Tofa A.I., Kamara A.Y., Babaji B.A., Aliyu K.T., Ademulegun T.D., Bebeley J.F. (2022). Maize yield as affected by the interaction of fertilizer nitrogen and phosphorus in the Guinea savanna of Nigeria. Heliyon.

[68] Krishnaprabu S. (2020). Impact of integrated nutrient management practices on distribution of nitrogen fractions and nitrogen use efficiency by maize crop in soil. Plant Archives.

[69] Sravan U.S., Singh S.P., Neupane M.P. (2021). Response of basmati rice varieties to integrated nutrient management. J. Plant Nutr..

[70] Fazily T., Thakral S., Dhaka A., Sharma M. (2020). Effect of integrated nutrient management on fertilizer use efficiency in wheat (Triticum aestivum L.) under irrigated condition. International Journal of Advances in Agricultural Science and Technology.

[71] Rex Immanuel R., Rao G.B.S., Perumal M.S., Arivukkarasu K., Mullaivendhan V. (2021). Nutrient uptake and use efficiency of maize (Zea Mays L.) as influenced by microbial seed inoculation, NPK fertilization and panchagavya foliar application. Plant Archives.

[72] Dubey R.K., Dubey P.K., Abhilash P.C. (2019). Sustainable soil amendments for improving the soil quality, yield and nutrient content of Brassica juncea (L.) grown in different agroecological zones of eastern Uttar Pradesh, India. Soil Tillage Res..

[73] Tahir M., Khan A.H., Batool M., Zeeshan H.M., Iqbal M., Khan A.G. (2012). Effect of dairy manure and rice planting methods on yield, soil quality, water-use efficiency, and economics of rice and succeeding wheat crop. Commun. Soil Sci. Plant Anal..

[74] Demelash N., Bayu W., Tesfaye S., Ziadat F., Sommer R. (2014). Current and residual effects of compost and inorganic fertilizer on wheat and soil chemical properties. Nutrient Cycl. Agroecosyst..

[75] ur Rehman M.Z., Zafar M., Waris A.A., Rizwan M., Ali S., Sabir M., Usman M., Ayub M.A., Ahmad Z. (2020). Residual effects of frequently available organic amendments on cadmium bioavailability and accumulation in wheat. Chemosphere.

[76] Manzoor M., Shafi M., Sohail A., Ali S., Anwar S., Fahad S. (2019). Fertilizer management for improving water use efficiency in winter cereals in semiarid region of northwest, Pakistan. Russ. Agric. Sci..

[77] Wang X., Yan J., Zhang X., Zhang S., Chen Y. (2020). Organic manure input improves soil water and nutrients use for sustainable maize (Zea mays. L) productivity on the Loess Plateau. PLoS One.

[78] Mohamed E.M., Watthier M., Zanuncio J.C., Santos R.H.S. (2017). Dry matter accumulation and potato productivity with green manure. IDESIA.

[79] Adekiya A.O., Agbede T.M., Aboyeji C.M., Dunsin O., Simeon V.T. (2019). Effects of biochar and poultry manure on soil characteristics and the yield of radish. Sci. Hortic..

[80] Oladele S., Adeyemo A., Awodun M., Ajayi A., Fasina A. (2019). Effects of biochar and nitrogen fertilizer on soil physicochemical properties, nitrogen use efficiency and upland rice (Oryza sativa) yield grown on an Alfisol in Southwestern Nigeria. Int. J. Recycl. Org. Waste Agric..

[81] Seleiman M.F., Abdelaal M.S. (2018). Effect of organic, inorganic and bio-fertilization on growth, yield and quality traits of some chickpea (Cicer arietinum L.) varieties. Egypt. J. Agron..

[82] Peralta-Antonio N., Watthier M., Silva Santos R.H. (2021). Green manure and mineral fertilizer in sequential cropping: effect on dry matter, yield, accumulation and recovery efficiency of nutrients. Commun. Soil Sci. Plant Anal..

[83] Huang M., Fan L., Chen J., Jiang L., Zou Y. (2018). Continuous applications of biochar to rice: effects on nitrogen uptake and utilization. Sci. Rep..

[84] Mitran T., Mani P.K. (2017). Effect of organic amendments on rice yield trend, phosphorus use efficiency, uptake, and apparent balance in soil under long-term rice-wheat rotation. J. Plant Nutr..

[85] Huang M., Shan S., Xie X., Zhou X., Zou Y., Uphoff N. (2019). Grain yield and nitrogen utilization in response to reducing nitrogen rate in hybrid rice transplanted as single seedlings. Exp. Agric..

[86] Mamedov A.I., Bar-Yosef B., Levkovich I., Rosenberg R., Silber A., Fine P., Levy G.J. (2014). Amending soil with sludge, manure, humic acid, orthophosphate and phytic acid: effects on aggregate stability. Soil Res..

[87] Sayara T., Basheer-Salimia R., Hawamde F., Sánchez A. (2020). Recycling of organic wastes through composting: process performance and compost application in agriculture. Agronomy.

[88] Noori Z., Delavar M.A., Safari Y., Alavi-Siney S.M. (2021). Reclamation of a calcareous sodic soil with combined amendments: interactive effects of chemical and organic materials on soil chemical properties. Arabian J. Geosci..

[89] Okebalama C.B., Safo E.Y., Yeboah E., Abaidoo R.C., Logah V. (2019). Vegetative and reproductive performance of maize to nitrogen and phosphorus fertilizers in Plinthic Acrisol and Gleyic Plinthic Acrisol. J. Plant Nutr..

[90] Owino J., Wandiga S., Olago D., Oghaiki A.N. (2020).

[91] Ahozonlin M.C., Dossa L.H. (2020). Diversity and resilience to socio-ecological changes of smallholder lagune cattle farming systems of Benin. Sustainability.

[92] Deng T., Wang J.-H., Gao Z., Shen S., Liang X.-G., Zhao X., Chen X.-M., Wu G., Wang X., Zhou S.-L. (2023). Late split-application with reduced nitrogen fertilizer increases yield by mediating source–sink relations during the grain filling stage in summer maize. Plants.

