# Supplementary material for: Hill-placement of manure and fertilizer for improving maize nutrient- and water-use efficiencies in the northern Benin
Source: Heliyon. 2023 Jul 8;9(7):e17823. doi: 10.1016/j.heliyon.2023.e17823 (PMC10362180; doi:10.1016/j.heliyon.2023.e17823)
Supplement: Multimedia component 1 [file mmc1.docx]

# SUPPLEMENTAL MATERIAL

**Figure S1:** Comparison between daily cumulative rainfall and drainage for DSSAT-CERES Maize for the calibration year (2014) by Tovihoudji et al. (2019) and recalibrated in this study.

|  | **NM** | **3M** | **6M** |  |
| --- | --- | --- | --- | --- |
| **LAI (m^2^ m^-2^)** |  |  |  | **2015** |
|  |  |  |  | **2014** |
|  |  |  |  | **2013** |
|  |  |  |  | **2012** |
|  | Days After Sowing (DAS) | | |  |

**Figure S2**: Effect of hill-placed manure and fertilizer on leaf area index of maize plant at various measurement dates, over four growing seasons (2012-2015). NM: no manure, 3M: 3 t ha^-1^ of applied manure, 6M: 6 t ha^-1^ of applied manure. NF: no fertilizer, 50F: half of fertilizer recommended application rate, 100F: Full fertilizer recommended application rate. Error bars represents the standard deviation. Data from the 3M treatments are missing in 2014 and 2015.

|  | **NM** | **3M** | **6M** |  | |  |
| --- | --- | --- | --- | --- | --- | --- |
| **TDM (g m^2^)** |  |  |  | **2015** | |  |
|  |  |  |  | **2014** | |  |
|  |  |  |  | **2013** | |  |
|  |  |  |  | **2012** | |  |
|  | Days After Sowing (DAS) | | | |  | |

**Figure S3:** Effect of hill-placed manure and fertilizer on total dry matter of maize plant at various measurement dates, over four growing seasons (2012-2015). NM: no manure, 3M: 3 t ha^-1^ of applied manure, 6M: 6 t ha^-1^ of applied manure. NF: no fertilizer, 50F: half of fertilizer recommended application rate, 100F: Full fertilizer recommended application rate. Error bars represents the standard deviation. Data from the 3M treatments are missing in 2014 and 2015.

|  | **NM** | **3M** | **6M** |  |
| --- | --- | --- | --- | --- |
| **SWS (mm)** |  |  |  | **2015** |
|  |  |  |  | **2014** |
|  |  |  |  | **2013** |
|  |  |  |  | **2012** |
|  | Days After Sowing (DAS) | Days After Sowing (DAS) | Days After Sowing (DAS) |  |

**Figure S4:** Effect of hill-placed manure and fertilizer on water stock in the soil profile (0-60cm) of maize plant over four growing seasons (2012-2015). NM: no manure, 3M: 3 t ha^-1^ of applied manure, 6M: 6 t ha^-1^ of applied manure. NF: no fertilizer, 50F: half of fertilizer recommended application rate, 100F: Full fertilizer recommended application rate. Error bars represents the standard deviation. Data from the 3M treatments are missing in 2014 and 2015.

**Supplemental Table 1:** Summary of the general analysis of variance for all variables.

| Variables | Source of variation (*p-values*) | | | | | | |
| --- | --- | --- | --- | --- | --- | --- | --- |
|  | Y | M | F | Y × M | Y × F | M × F | Y × M × F |
| LAI max | ***<0.001*** | ***<0.001*** | ***<0.001*** | *0.783* | ***<0.001*** | ***<0.001*** | ***0.004*** |
| TDM at flowering stage | ***<0.001*** | ***<0.001*** | ***<0.001*** | *0.999* | *0.905* | *0.301* | *0.999* |
| SWS at flowering stage | ***<0.001*** | *0.223* | *0.88* | *0.083* | *0.923* | *0.27* | *0.24* |
| ETR | ***<0.001*** | ***0.015*** | *0.111* | ***<0.001*** | *0.126* | *0.246* | *0.842* |
| WUE-grain | ***<0.001*** | ***<0.001*** | ***<0.001*** | ***<0.001*** | ***<0.001*** | ***0.002*** | ***<0.001*** |
| WUE-TDM | ***<0.001*** | ***<0.001*** | ***<0.001*** | ***<0.001*** | ***<0.001*** | ***<0.001*** | ***0.004*** |
| RUE | ***<0.001*** | ***<0.001*** | ***<0.001*** | ***<0.001*** | ***0.017*** | ***<0.001*** | ***<0.001*** |
| N uptake | ***<0.001*** | ***<0.001*** | ***<0.001*** | ***<0.001*** | ***0.003*** | ***0.022*** | *0.168* |
| P uptake | ***<0.001*** | ***<0.001*** | ***<0.001*** | ***<0.001*** | ***0.002*** | ***<0.001*** | *0.053* |
| K uptake | ***<0.001*** | ***<0.001*** | ***<0.001*** | ***<0.001*** | *0.412* | *0.164* | *0.646* |
| N balance | ***<0.001*** | ***<0.001*** | ***<0.001*** | ***<0.001*** | ***0.003*** | ***0.022*** | *0.168* |
| P balance | ***<0.001*** | ***<0.001*** | ***<0.001*** | ***<0.001*** | ***0.002*** | ***0.001*** | *0.053* |
| K balance | ***<0.001*** | *0.085* | *0.163* | ***<0.001*** | *0.412* | *0.164* | *0.646* |
| NUEre-N | ***<0.001*** | ***<0.001*** | ***<0.001*** | ***0.002*** | *0.406* | ***<0.001*** | *0.331* |
| NUEre-P | ***0.036*** | ***<0.001*** | ***0.002*** | ***0.030*** | *0.977* | ***0.001*** | *0.823* |
| NUEre-K | ***<0.001*** | ***<0.001*** | ***<0.001*** | ***0.019*** | *0.224* | ***<0.001*** | *0.891* |
| NUEie-N | ***<0.001*** | *0.643* | *0.097* | ***<0.001*** | *0.907* | *0.448* | *0.114* |
| NUEie-P | ***<0.001*** | ***<0.001*** | *0.558* | ***<0.001*** | *0.737* | *0.630* | *0.274* |
| NUEie-K | ***<0.001*** | *0.102* | *0.058* | *0.128* | *0.826* | *0.141* | *0.578* |

Y: year, M: manure, F: fertilizer, DAS: days after sowing, LAI: leaf area index, TDM: total dry matter, SWS: soil water storage, ETR: evapotranspiration, WUE: water use efficiency, NUEre: recovery efficiency, NUEie: internal utilization efficiency.
